# *GCK* Gene-Body Hypomethylation Is Associated with the Risk of Coronary Heart Disease

**DOI:** 10.1155/2014/151723

**Published:** 2014-02-17

**Authors:** Limin Xu, Dawei Zheng, Lingyan Wang, Danjie Jiang, Haibo Liu, Leiting Xu, Qi Liao, Lina Zhang, Panpan Liu, Xinbao Shi, Zhaoyang Wang, Lebo Sun, Qingyun Zhou, Ni Li, Yi Huang, Yanping Le, Meng Ye, Guofeng Shao, Shiwei Duan

**Affiliations:** ^1^Zhejiang Provincial Key Laboratory of Pathophysiology, Ningbo University School of Medicine, Ningbo, Zhejiang 315211, China; ^2^The Affiliated Hospital, Ningbo University School of Medicine, Ningbo, Zhejiang 315211, China; ^3^Ningbo Medical Center Lihuili Hospital, Ningbo University School of Medicine, Ningbo, Zhejiang 315041, China; ^4^Bank of Blood Products, Ningbo No. 2 Hospital, Ningbo, Zhejiang 315010, China; ^5^Yinzhou People's Hospital, Ningbo University School of Medicine, Ningbo, Zhejiang 315040, China

## Abstract

*Objectives*. Glucokinase encoded by *GCK* is a key enzyme that facilitates phosphorylation of glucose to glucose-6-phosphate. Variants of *GCK* gene were shown to be associated with type 2 diabetes (T2D) and coronary heart disease (CHD). The goal of this study was to investigate the contribution of *GCK* gene-body methylation to the risk of CHD. *Design and Methods*. 36 patients (18 males and 18 females) and 36 age- and sex-matched controls were collected for the current methylation research. DNA methylation level of the CpG island (CGI) region on the *GCK* gene-body was measured through the sodium bisulfite DNA conversion and pyrosequencing technology. *Results.* Our results indicated that CHD cases have a much lower methylation level (49.77 ± 6.43%) compared with controls (54.47 ± 7.65%, *P* = 0.018). In addition, *GCK* gene-body methylation was found to be positively associated with aging in controls (*r* = 0.443, *P* = 0.010). *Conclusions*. Our study indicated that the hypomethylation of *GCK* gene-body was significantly associated with the risk of CHD. Aging correlates with an elevation of *GCK* methylation in healthy controls.

## 1. Introduction

Diabetes and cardiovascular disease (CVD) are two common diseases with close relationship [1]. At least 65% of diabetic patients died from heart diseases or stroke. Even when glucose levels are under control, diabetes would greatly increase twofold of the risk of heart disease and stroke [2]. As a major type of CVD, coronary heart disease (CHD) has drawn a lot of attention in its pathogenic mechanism. Evidence has shown that variation in CHD incidence and mortality among different populations may be explained by the interaction between environmental and genetic factors [3]. Environmental factors contributing to the development of CHD include diabetes, hypertension, plasma lipid, smoking, and obesity [4, 5].

Aberrant epigenetic modifications may bridge the environmental and genetic factors and thus lead to pathological consequences such as diabetes and CVD [6]. Familial aggregation of CHD, diabetes, and obesity can reflect epigenetic processes that can be explained by common environmental factors such as diet habits [3, 7]. By introducing or removing CpG sites, some single nucleotide polymorphisms (SNPs) may impact gene expression through the alteration of DNA methylation level and contribute to the risk of disease [8].

DNA methylation is an important modification of epigenetics and often occurs at CpG dinucleotides in mammals [9]. Hypermethylation in promoter regions has been known to repress gene expression [10, 11]. However, DNA methylation in gene-body shows a bell-shaped correlation with gene expression [12, 13]. Specifically, genes with middle-level expression have higher levels of gene-body methylation in plants and invertebrates [13]. Positive correlation between expression and gene-body methylation is also presented in a variety of human tissue types [12, 14]. Adrian Bird proposed that gene-body methylation represented “orphan promoters” that might be used at the early stages of development [15]. Methylation of gene-body may activate the transcription of canonical gene, although the functions of the gene-body CGI remain largely unknown [11].

As a candidate gene of type 2 diabetes (T2D) [16], *GCK* encoded glucokinase that is a key enzyme of glucose phosphorylation [17, 18]. Studies have shown that the occurrence of cardiovascular morbidity and mortality is positively associated with the plasma glucose levels [19, 20]. Glucokinase is able to facilitate hepatic glucose uptake during hyperglycemia and determines the threshold for glucose-stimulated insulin secretion [17, 21–23]. The specific molecular mechanisms underlying the progression to hyperglycemic states remain uncertain. A *GCK* promoter variant (−30G>A, rs1799884) was shown to increase the risk of both T2D [24, 25] and CHD [26, 27]. Hypermethylation of hepatic *GCK* promoter in aging rats contributes to diabetogenic potential [28]. Human *GCK *CpG island (CGI) is located in the gene-body but not near promoter. Here, we performed an association study of *GCK* gene-body methylation with the risk of CHD in a well matched case-control cohort.

## 2. Materials and Methods

### 2.1. Samples and Clinical Data

36 CHD patients (18 males and 18 females) and 36 age- and sex-matched controls were collected from Ningbo Lihuili Hospital. The details of the inclusion criteria were presented in our previous publication [29]. All the collected individuals were Han Chinese from Ningbo city in eastern China. All of the experiments were approved by the Ethical Committee of Ningbo Lihuili Hospital, and written informed consent was obtained from all the subjects.

### 2.2. Biochemical Analyses

Nucleic acid extraction analyzer (Lab-Aid 820, Xiamen, China) was used to extract genomic DNA from peripheral blood samples. The concentrations of extracted DNA were measured by the ultramicro nucleic acid ultraviolet tester (NANODROP 1000, Wilmington, USA). Plasma levels of biochemical factors (including TG, TC, HDL, LDL, ApoA 1, ApoB, ApoE, Lp(a), hs-CRP, ALB, GLB, ALT, AST, ALP and GGT) were measured using the methods described in our previous study [30]. DNA methylation was measured using the sodium bisulphite DNA conversion coupled with pyrosequencing [30]. Genomic DNA was chemically modified by sodium bisulfite (EpiTech Bisulfite Kits; Qiagen) to convert all unmethylated cytosines to uracils while the methylated cytosines unchanged. The bisulfite converted DNA and the polymerase chain reaction (PCR) primers which were designed by PyroMark Assay Design software were mixed and performed with PCR (Pyromark PCR Kit; Qiagen). The PCR products were degenerated and released to single strand products for pyrosequencing and the sequencing was conducted by Q24 machine and reagents (Pyromark Gold Q24 Reagents; Qiagen). The forward primer sequence was 5′-TGGATGGTTTAGTGTATAAGTTGTATT-3′. The reverse primer sequence was 5′-Biotin-CACCTCATCCTCCACATTCAT-3′, and the sequencing primer sequence was 5′-AAGTGGGGTTTAAAAAG-3′.

### 2.3. Statistical Analyses

Statistical analyses were performed using the SPSS package (version 16.0) to investigate the association of *GCK* methylation with CHD and various biochemical factors. Values of biochemical indicators deviated from normality were corrected via a logarithmic transformation. A more conservative nonparametric approach was used for data which were unable to be normalized. The correlations of DNA methylation with age and biochemical indicators were performed using SPSS package and R statistical software. All the *P* values were adjusted for the history of age, smoking, diabetes, and hypertension. Methylation levels were presented as means ± SD. The adjusted calculation was analyzed by SPSS package with binary logistic regression. A two-tailed *P* < 0.05 was considered to be significant.

## 3. Results

**Figure 1:**
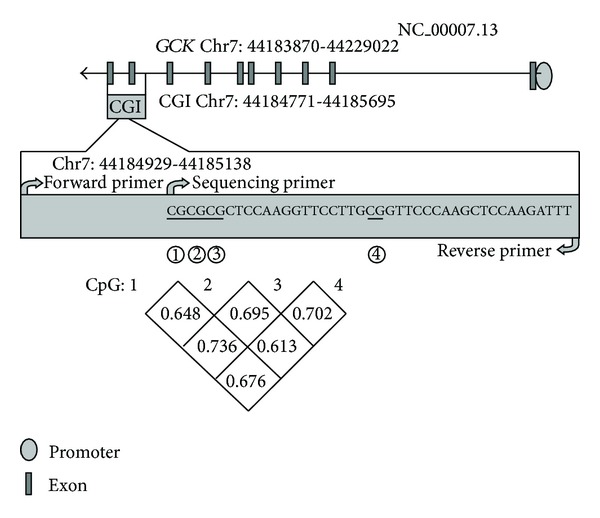
The four tested CpG sites in *GCK* gene.

**Figure 2:**
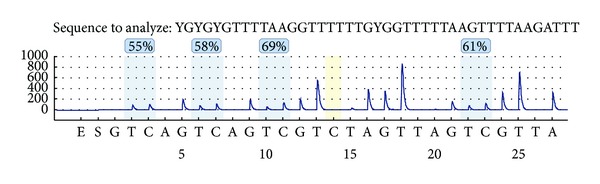
The methylation levels of four CpG sites on *GCK* gene-body of one sample.

Two CGIs of *GCK* gene (hg19, chr7: 44183870-44229022) were located in the gene-body. One of the CGIs was too short (223 bp) to design the primers for bisulfite pyrosequencing. Therefore, the other CGI was used for the methylation assay. As shown in Figure 1, this CGI spans exon 9 and part of exon 10 (hg19, chr7: 44184771-44185695). DNA methylation percentages of four CpG sites were obtained using the bisulfite pyrosequencing assay on a 210 bp fragment of intron 9 and exon 9 (hg19, chr7: 44184929-44185138). Significant correlation of the DNA methylation levels was observed among the four CpGs (Figure 1, *r* = 0.6–0.8, *P* < 0.0001). The methylation levels of the four CpG sites on *GCK* gene-body were measured using the Pyromark Q24 instrument (Figure 2). In our study, the raw data of methylation levels of the four CpG sites on *GCK* gene-body were presented in Supplemental Table  1 (See Supplementary Material available at http://dx.doi.org/10.1155/2014/151723). Mean methylation level and each of the four CpG sites were used to compare the differences between cases and controls, between males and females, and the relationship between *GCK* methylation and biochemical indicators.

**Figure 3:**
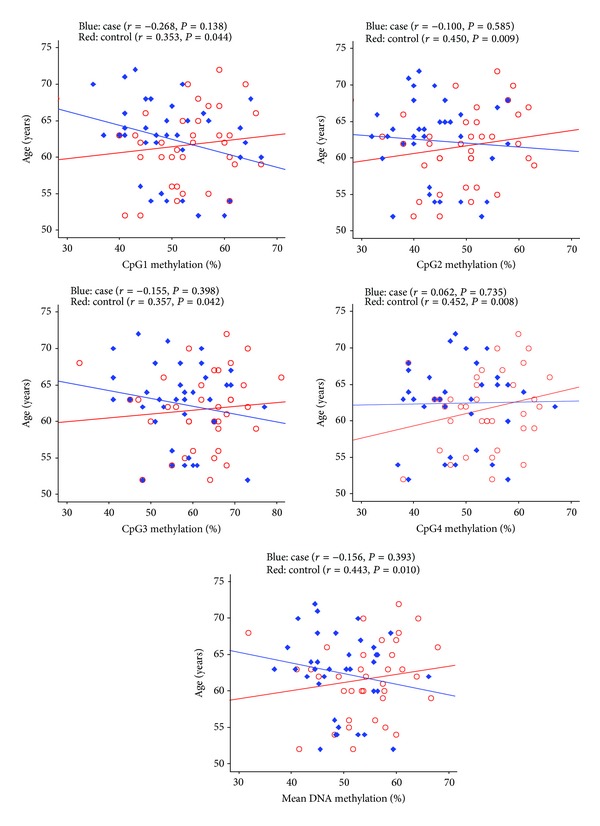
Correlation between *GCK* gene DNA methylation and age. The *P* values were adjusted by age, history of smoking, diabetes, and hypertension.

As shown in Table 1 and Supplemental Figure  1, significant difference was observed between CHD cases and healthy controls. CHD cases have a significantly lower methylation level (49.77 ± 6.43%) compared with controls (54.47 ± 7.65%, *P* = 0.018). The similar trend was observed in three CpGs (Table 1 and Supplemental Figure  2: CpG2: 43.42 ± 7.93% versus 49.42 ± 8.26%, *P* = 0.005; CpG3: 56.81 ± 8.74% versus 62.06 ± 9.58%, *P* = 0.036; CpG4: 48.86 ± 7.44% versus 53.67 ± 7.11%, *P* = 0.031). There was no difference of *GCK* methylation level between males and females and no significant interaction between gender and disease (Table 1, *P* > 0.05). A significant difference of the CpG2 methylation level with CHD was observed in males (Table 2, *P* = 0.030). Mean *GCK* methylation was associated with aging in controls (*r* = 0.443, *P* = 0.010; Figure 3). No gender-specific difference was found for the association between *GCK *methylation and age (data not shown).

We explored the correlation of these biochemical indicators and DNA methylation. As shown in Supplemental Table  2, significant associations were observed for triglyceride (TG, CpG2: *P* = 0.044; CpG3: *P* = 0.019), low-density lipoprotein (LDL, CpG3: *P* = 0.029), apolipoprotein B (ApoB, CpG2: *P* = 0.048), alanine amiotransferase (ALT, CpG2: *P* = 0.044), and *γ*-glutamyl transpeptidase (GGT, CpG1: *P* = 0.020; CpG2: *P* = 0.039, CpG3: *P* = 0.006, CpG2: *P* = 0.033).

## 4. Discussion

Type 2 diabetes (T2D) patients often have high risk of developing cardiovascular diseases including CHD [31]. We hypothesize that *GCK* gene as a candidate diabetic gene may also contribute to the risk of CHD. Our results revealed that CHD cases have a hypomethylation level in *GCK* gene-body compared with controls. In addition, average *GCK* methylation level elevated along with increasing age in controls.

The methylation levels of both promoter and gene-body are highly dynamic [32]. DNA methylation causes gene silencing through blocking the binding of transcription factors or methyl-binding proteins [33]. Genome-wide study of DNA methylation has identified that aberrant methylation of gene bodies was able to contribute to the risk of heart failure [34]. DNA methylation marks on gene bodies were shown to regulate gene expression [11]. Positive correlation between gene-body methylation and gene expression was presented in a variety of human tissue types [12, 14]. Gene-body methylation was shown to play an important role in regulating cell context-specific alternative promoters in human and mouse tissues [35].

In the present study, we found that there was no specific CGI in the human *GCK* promoter. Evidence has shown that transcribed regions tend to have higher levels of methylation than intergenic or promoter regions [13, 36]. Hypermethylation of gene-body methylation often indicates a higher level of gene expression in human tissue and cell types [13, 35]. It can slow down the transcription elongation and suppress the initiation of abnormal transcription [37]. Gene-body methylation represented “orphan promoters” that might be used at the early stages of development [15]. DNA methylation can cause chromatin structure changes of the corresponding region in the genome and change the restriction endonuclease sites [38]. Thereby we speculate that the decrease of *GCK* methylation in CHD cases may influence the transcription and gene expression.

The rat study has shown that *GCK *gene methylation is important in the early development [39]. Reduced *GCK* expression was shown in aged rats through the elevation of *GCK *gene promoter methylation [28, 40]. Rat *GCK *mRNA in hepatocytes increased 4-fold upon treatment with 5-Aza-CdR, a cytosine methylation inhibitor [28]. Downregulation of *GCK* expression was reversed by the 5-Aza-CdR in the cell model of steatosis [40].

Differences of age-dependent methylation have been observed in multiple independent studies [41–43]. Aging induces global and complex changes of DNA methylation in humans [42]. Global methylation levels become more variable between monozygotic twins during their lifetime [44]. Age-dependent gene hypermethylation was also observed in human skin tissue [45]. In the present study, we also found a significant association between *GCK* gene-body hypermethylation and aging in healthy individuals.

Our study confirmed that human *GCK* gene-body methylation level was positively correlated with ageing in the healthy controls. We speculated that *GCK *hypermethylation might play an important role to protect people from cardiovascular diseases, although the exact mechanisms need to be clarified in the future study.

There are some limitations of our results to be taken with caution. Firstly, we explored only a part of CGI. Our findings of these four CpGs may not represent the whole CGI of *GCK* gene. Secondly, the sample size of our study is small. Validations of our findings are needed in larger samples and in other ethnic populations. Last but not least, we cannot exclude a chance that variations in the administrated drugs and diets may affect our findings on *GCK *methylation, although our results have been employed with a strict adjustment by multiple factors including hypertension, smoking habit, and diabetes.

## 5. Conclusion

In conclusion, our results found that lower methylation level of* GCK* gene-body was associated with the risk of CHD in Chinese. Our results also confirmed that *GCK* methylation level had a positive correlation with aging in humans.

## Supplementary Material

Supplemental Table 1: The raw data of methylation levels of the four CpG sites on *GCK* gene body.
Supplemental Table 2: Correlation between *GCK* gene DNA methylation and biochemical indicators in cases.
Supplemental Figure 1*：*Comparison of *GCK* gene mean DNA methylation levels within subgroups and gender separately.
Supplemental Figure 2: Four CpG sites DNA methlation levels in cases and controls.

## Figures and Tables

**Table 1:** Comparison of* GCK* gene DNA methylation levels within subgroups and gender separately.

DNA methylation (%)	Case	Control	Subgroup *P *	Male	Female	Gender *P *	Gender ∗ subgroup Interaction *P *
CpG1	50.00 ± 8.34	52.72 ± 8.25	0.232	52.11 ± 8.01	50.61 ± 8.73	0.955	0.556
CpG2	43.42 ± 7.93	49.42 ± 8.26	0.005*	46.14 ± 9.16	46.69 ± 8.10	0.932	0.668
CpG3	56.81 ± 8.74	62.06 ± 9.58	0.036*	59.92 ± 9.04	58.94 ± 10.00	0.887	0.909
CpG4	48.86 ± 7.44	53.67 ± 7.11	0.031*	50.83 ± 7.48	51.69 ± 7.83	0.258	0.667
Mean	49.77 ± 6.43	54.47 ± 7.65	0.018*	52.25 ± 7.23	51.99 ± 7.67	0.842	0.658

The *P* values were adjusted by age, history of smoking, diabetes, and hypertension.

*P < 0.05.

**Table 2:** Comparison of* GCK* gene DNA methylation levels between cases and controls in male and female separately.

DNA methylation (%)	Male			Female		
	Case	Control	*P *	Case	Control	*P *
CpG1	50.17 ± 8.50	54.06 ± 7.19	0.321	49.83 ± 8.42	51.39 ± 9.20	0.674
CpG2	42.72 ± 9.52	49.56 ± 7.57	0.030*	44.11 ± 6.15	49.28 ± 9.12	0.067
CpG3	57.17 ± 8.99	62.67 ± 8.46	0.155	56.44 ± 8.73	61.44 ± 10.79	0.138
CpG4	48.06 ± 7.01	53.61 ± 7.06	0.085	49.67 ± 7.96	53.72 ± 7.36	0.228
Mean	49.53 ± 6.58	54.97 ± 6.98	0.063	50.01 ± 6.45	53.96 ± 8.44	0.168

The *P* values were adjusted by age, history of smoking, diabetes, and hypertension.

*P < 0.05.

## Abbreviations

*GCK*: Glucokinase
CHD: Coronary heart disease
CVD: Cardiovascular disease
GWAS: Genome-wide association studies
T2D: Type 2 diabetes
CGI: CpG island
LDL: Low density lipoprotein
TG: Triglyceride
ApoB: Apolipoprotein B
ALT: Alanine amiotransferase
GGT: *γ*-Glutamyl transpeptidase.
