# Supplementary material for: GCK Gene-Body Hypomethylation Is Associated with the Risk of Coronary Heart Disease
Source: Biomed Res Int. 2014 Feb 17;2014:151723. doi: 10.1155/2014/151723 (PMC3947703; doi:10.1155/2014/151723)
Supplement: Supplementary file 1 — Supplemental Table 1: The raw data of methylation levels of the four CpG sites on GCK gene body. Supplemental Table 2: Correlation between GCK gene DNA methylation and biochemical indicators in cases. Supplemental Figure 1：Comparison of GCK gene mean DNA methylation levels within subgroups and gender separately. Supplemental Figure 2: Four CpG sites DNA methlation levels in cases and controls. [file 151723.f1.pdf]

Supplemental Table 1: The raw data of methylation levels of the four CpG sites on GCK gene body

No.	Age	Gender	Group	Smoking	Hypertension	Diabetes	CpG1	CpG2	CpG3	CpG4	Mean
1	52	1	1	0	1	1	55	53	73	58	60
2	54	1	1	0	1	0	61	54	60	37	53
3	54	1	1	1	0	0	52	49	61	55	54
4	56	1	1	1	0	0	44	43	55	52	49
5	60	1	1	1	0	0	67	34	65	58	56
6	61	1	1	1	0	0	52	21	58	51	46
7	62	1	1	0	1	1	45	38	48	42	43
8	62	1	1	1	1	0	47	40	53	46	47
9	63	1	1	1	1	0	41	40	58	40	45
10	63	1	1	0	1	0	37	32	41	38	37
11	63	1	1	1	1	0	51	49	52	51	51
12	64	1	1	0	1	0	45	41	49	46	45
13	65	1	1	1	0	1	57	45	68	56	57
14	66	1	1	0	1	0	41	33	41	43	40
15	67	1	1	0	1	0	50	56	69	39	54
16	68	1	1	1	1	0	65	58	62	52	59
17	70	1	1	0	0	0	52	44	62	54	53
18	71	1	1	1	1	0	41	39	54	47	45
19	52	2	1	0	1	0	60	36	48	39	46
20	54	2	1	0	0	0	49	45	55	46	49
21	54	2	1	0	1	0	46	44	58	48	49
22	55	2	1	1	0	0	48	43	59	47	49
23	60	2	1	0	0	0	63	55	51	58	57

24	62	2	1	0	1	1	64	58	77	67	67
25	63	2	1	0	1	0	47	42	56	45	48
26	63	2	1	0	1	0	49	49	64	44	52
27	63	2	1	0	1	1	40	35	45	44	41
28	64	2	1	0	0	0	63	42	58	61	56
29	64	2	1	0	1	0	41	36	60	39	44
30	65	2	1	0	1	0	53	47	69	58	57
31	65	2	1	0	0	0	49	46	57	53	51
32	66	2	1	1	0	0	56	49	63	56	56
33	68	2	1	0	0	0	45	46	51	39	45
34	68	2	1	0	0	0	46	40	57	52	49
35	70	2	1	0	0	1	35	40	41	50	42
36	72	2	1	0	1	1	43	41	47	48	45
37	52	1	0	1	0	0	41	40	48	38	42
38	54	1	0	1	0	0	51	41	55	47	49
39	55	1	0	1	1	0	52	45	58	50	51
40	56	1	0	0	0	0	51	52	66	56	56
41	59	1	0	1	0	0	62	42	66	61	58
42	59	1	0	1	0	0	67	63	75	63	67
43	60	1	0	1	0	0	61	51	66	54	58
44	60	1	0	0	1	0	52	51	65	47	54
45	62	1	0	0	0	0	54	49	66	49	55
46	62	1	0	0	0	0	48	43	56	50	49
47	63	1	0	1	0	0	59	55	69	63	62
48	63	1	0	1	1	0	43	43	45	45	44
49	65	1	0	0	0	1	52	50	62	52	54

50	65	1	0	1	1	0	55	52	72	59	60
51	66	1	0	1	1	1	45	38	53	52	47
52	67	1	0	1	0	0	57	62	65	57	60
53	70	1	0	1	1	0	64	59	73	62	65
54	72	1	0	0	1	1	59	56	68	60	61
55	52	2	0	0	0	0	44	45	64	55	52
56	54	2	0	0	1	0	61	51	68	61	60
57	55	2	0	0	0	0	57	56	65	55	58
58	56	2	0	0	0	0	50	50	60	45	51
59	60	2	0	0	0	0	44	45	59	53	50
60	60	2	0	0	1	0	50	62	50	54	54
61	60	2	0	0	0	0	48	45	59	55	52
62	61	2	0	0	0	0	51	51	68	61	58
63	62	2	0	0	1	1	44	38	54	46	46
64	62	2	0	0	0	0	60	60	73	64	64
65	63	2	0	0	1	0	61	53	69	52	59
66	63	2	0	0	1	0	54	51	62	47	54
67	63	2	0	0	0	1	40	34	47	44	41
68	66	2	0	0	1	0	66	60	81	66	68
69	67	2	0	0	0	1	59	52	66	53	58
70	68	2	0	0	1	1	28	28	33	39	32
71	68	2	0	0	0	0	55	58	69	61	61
72	70	2	0	0	1	0	53	48	59	56	54

Gender: 1 = Male, 2 = Female; Group: 1 = Case, 0 = Control; Smoking: 1 = Yes, 0 = No; Hypertension: 1 = Yes, 0 = No; Diabetes: 1 = Yes, 0 = No; Methylation levels of the four CpG sits and mean levels were presented in the form of percentage (%).

Supplemental Table 2: Correlation between *GCK* gene DNA methylation and biochemical indicators in cases

Case	CpG1		CpG2		CpG3		CpG4	
correlation with methylation	r	*P*	r	*P*	r	*P*	r	*P*
TG	-0.339	0.062	-0.364	0.044*	-0.420	0.019*	-0.286	0.119
TC	-0.295	0.107	-0.345	0.057	-0.229	0.215	-0.166	0.371
HDL	-0.170	0.361	-0.140	0.451	-0.015	0.934	0.020	0.915
LDL	0.146	0.434	0.078	0.678	0.392	0.029*	0.229	0.215
ApoA	-0.096	0.609	-0.071	0.705	0.085	0.650	-0.099	0.598
ApoB	-0.295	0.107	-0.357	0.048*	-0.247	0.181	-0.240	0.194
ApoE	0.079	0.673	-0.065	0.730	0.161	0.387	0.114	0.541
Lp(a)	0.125	0.501	0.108	0.565	0.160	0.390	0.318	0.081
hs-CRP	-0.193	0.298	-0.224	0.226	-0.041	0.829	-0.244	0.187

ALB	-0.054	0.772	0.021	0.912	0.157	0.398	-0.172	0.356
GLB	0.243	0.187	0.202	0.275	0.231	0.211	-0.031	0.869
A/G	-0.299	0.102	-0.248	0.178	-0.199	0.284	-0.093	0.620
ALT	0.164	0.378	0.364	0.044*	0.307	0.093	-0.009	0.961
AST	0.192	0.301	0.320	0.079	0.266	0.147	0.218	0.239
ALP	-0.307	0.094	-0.118	0.526	0.122	0.512	-0.229	0.214
GGT	0.414	0.020*	0.373	0.039*	0.479	0.006*	0.384	0.033*

TG: triglyceride; TC: total cholesterol; HDL: high-density lipoprotein; LDL: low-density lipoprotein; ApoA1: apolipoprotein A1; ApoB: apolipoprotein B; ApoE: apolipoprotein E; Lp(a): lipoprotein(a); hs-CRP: high-sensitivity C-reactive protein; ALB: albumin; GLB: globulin; A/G: the ratio of albumin to globulin; ALT: alanine amiotransferase; AST: aspartate aminotransferase; ALP: alkaline phosphatase; GGT: γ-glutamyl transpeptadase

The *P* values were adjusted by age, history of smoking, diabetes and hypertension * $P < 0.05$
