# Supplementary material for: GCK Gene-Body Hypomethylation Is Associated with the Risk of Coronary Heart Disease
Source: Biomed Res Int. 2014 Feb 17;2014:151723. doi: 10.1155/2014/151723 (PMC3947703; doi:10.1155/2014/151723)
Supplement: Supplementary file 3 [file 151723.f3.pdf]

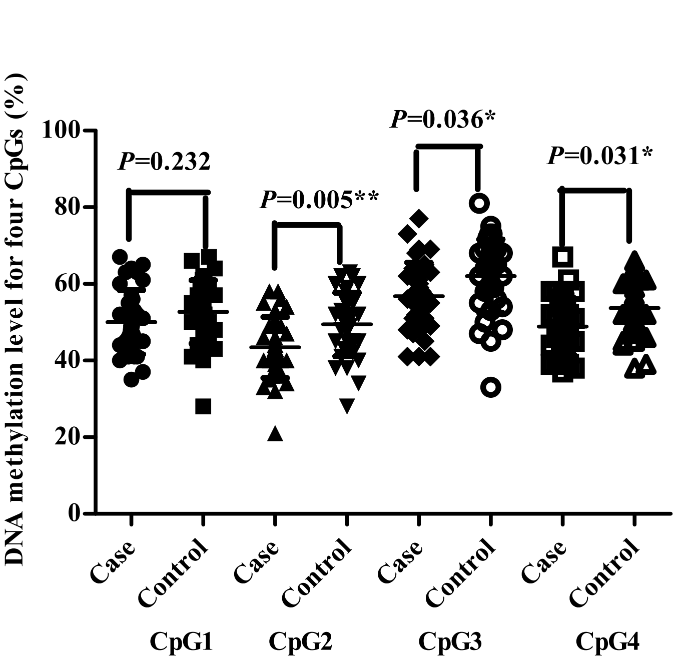

The methylation level was showed with mean± SD

The P values were adjusted by age, history of smoking, diabetes and hypertension

* $P < 0.05$ ** $P < 0.01$
